# Development of an Improved Menopausal Symptom-Alleviating Licorice (*Glycyrrhiza uralensis*) by Biotransformation Using *Monascus albidulus*

**DOI:** 10.4014/jmb.1909.09037

**Published:** 2019-11-22

**Authors:** Kang Uk Kim, Sung-Jin Lee, Inhyung Lee

**Affiliations:** 1 Department of Bio and Fermentation Convergence Technology, BK2 PLUS Project, Kookmin University, Seoul 02707, Republic of Korea; 2 Food R&D Center, SK Bioland Co., Ltd., Gyeonggi 15407, Republic of Korea

**Keywords:** *Monascus albidulus*, licorice, menopausal symptoms, liquiritigenin, estrogen receptor β, monacolin K

## Abstract

Licorice (*Glycyrrhiza uralensis*) contains several compounds that have been reported to alleviate menopausal symptoms via interacting with estrogen receptors (ERs). The compounds exist mainly in the form of glycosides, which exhibit low bioavailability and function. To bioconvert liquiritin and isoliquiritin, the major estrogenic compounds, to the corresponding deglycosylated liquiritigenin and isoliquiritigenin, respectively, licorice was fermented with *Monascus*, which has been demonstrated to deglycosylate other substances. The contents of liquiritigenin and isoliquiritigenin in *Monascus*-fermented licorice increased by 10.46-fold (from 38.03 μM to 379.75 μM) and 12.50-fold (from 5.53 μM to 69.14 μM), respectively, compared with their contents in non-fermented licorice. *Monascus*-fermented licorice exhibited 82.5% of the ERβ binding activity of that observed in the positive control (17 β-estradiol), whereas the non-fermented licorice exhibited 54.1% of the binding activity in an in vivo ER binding assay. The increase in the ERβ binding activity was associated with increases in liquiritigenin and isoliquiritigenin contents. Liquiritigenin acts as a selective ligand for ERβ, which alleviates menopausal symptoms with fewer side effects, such as heart disease and hypertension, compared with a ligand for ERα. In addition, *Monascus*-fermented licorice contained 731 mg/kg of monacolin K, one of the metabolites produced by *Monascus* that reduces serum cholesterol. Therefore, *Monascus*-fermented licorice is a promising material for the prevention and treatment of menopausal syndrome with fewer side effects.

## Introduction

Decreased levels of estrogens cause menopause syndrome in women after middle age. Menopausal symptoms, such as hot flushes, night sweats, depression, sleep disturbances, vaginal dryness, bone loss, and changes in cardiovascular and metabolic function, considerably affect the quality of life for middle-aged women [3, 33]. Hormone replacement therapy (HRT) that uses pharmaceutical estrogens has been adopted to treat menopausal symptoms [10]. However, according to the Women's Health Initiative (WHI), HRT potentially has side effects that could lead to breast cancer and heart attacks, although it can effectively alleviate menopausal symptoms [17]. Therefore, HRT is limited to treatment of menopausal symptoms with minimal doses of replaced hormones [7]. Currently, many women prefer natural substitutes, such as herbal preparations or nutraceuticals, which are considered generally safer compared with HRT. Phytoestrogens are plant compounds that are considered to act similar to estrogens based on their analogous chemical structures [7]. Isoflavones, lignans, and coumestans are the major phytoestrogens [29]. Particularly, isoflavones, such as genistein and S-equol from soy, have been reported to be effective in alleviating menopausal symptoms [1, 15]. However, the effectiveness of other phytoestrogens in the treatment of menopausal symptoms is yet to be evaluated. In addition, some reports have raised concerns over potential long-term side effects, such as stroke, heart disease, and breast cancer [3, 17, 33]. Therefore, currently, phytoestrogen has not been considered as a therapeutic agent.

Replaced hormones and phytoestrogens alleviate menopausal symptoms by binding to estrogen receptor alpha (ERα) and estrogen receptor beta (ERβ) [34, 36]. ERα and ERβ are very similar structurally; however, their ligand-binding domains are very different with regard to their selectivity to ligands [27]. ERα and ERβ exhibit entirely different functions [32]. Generally, when estrogen-like compounds bind selectively to ERβ, they can function as tumor suppressors; therefore, estrogen-like compounds that bind to ERβ can alleviate menopausal symptoms without the side effects associated with menopausal therapies, such as breast cancer [32].

Licorice represents one of the most important traditional herbal medicines in Korea, China, Japan, and East Asia in general [44]. Some studies have suggested beneficial health effects of licorice and its bioactive constituents [18]. Licorice root extracts are often consumed as dietary supplements by women at menopause [5, 18]. According to Boonmuen *et al*.(2016), licorice root extracts harbor diverse compounds, including ER agonists and antagonists [5, 18]. Particularly, liquiritigenin, isoliquiritigenin, calycosin, methoxychalcone, vestitol, and glycycoumarin are estrogen agonists. Among them, liquiritigenin, an aglycone form of liquiritin, is a well-known selective agonist for the tumor-suppressive ERβ [30]. Liquiritigenin is notably less stimulatory of genes promoting proliferation and motility when compared with 17 β-estradiol (E2) [12]. Licorice is derived from three medicinal plants, *Glycyrrhiza uralensis*, *Glycyrrhiza glabra*, and *Glycyrrhiza inflata*. Each species has different compound profiles, and some compounds are specific markers for each species [40]. The contents of ER agonists, such as liquiritin, isoliquiritin, and liquiritigenin, are significantly higher in *G. uralensis* than in *G. glabra* or *G. inflata*. However, the mean content of liquiritigenin, an ERβ marker compound, in *G. uralensis*, is very low [21, 39]. Hwang *et al*. (2015) reported the bioconversion of liquiritin into liquiritigenin using *Laetiporus sulphureus* to increase liquiritigenin content in licorice; however, their study focused on liquiritin and liquiritigenin contents, but not on the bioconversion of associated compounds, such as liquiritin apioside and isoliquiritin [16].

*Monascus* spp. have been applied in the bioconversion of glycosides into their corresponding aglycones to enhance their functionality and bioavailability [14, 24]. In addition, *Monascus* spp. produce several bioactive compounds, such as monacolin K and γ-aminobutyric acid (GABA), during fermentation [9, 23, 26], which could provide additional functionality to bioconversion effects. Particularly, monacolin K (also known as mevinolin or lovastatin) lowers blood cholesterol levels by inhibiting the activity of HMG-CoA reductase, a key enzyme in the cholesterol biosynthesis pathway [8]. Therefore, *Monascus*-fermented licorice could benefit women at menopause by enhancing cardiovascular functions.

In the present study, licorice was fermented with *Monascus* to enrich the preferred estrogen-like compounds that bind selectively to ERβ. Several estrogenic compounds, including liquiritin and liquiritin apioside, were bioconverted into their corresponding aglycones, which could exhibit relatively higher functional activity and bioavailability. *Monascus*-fermented licorice exhibited the high ERβ binding activity and contained monacolin K. Therefore, *Monascus*-fermented licorice is a promising and alternative functional material for the management of menopausal symptoms without the side effects associated with other common therapies.

## Materials and Methods

### Chemicals and Media

Monacolin K, citrinin, liquiritin, liquiritigenin, trifluoroacetic acid, acetic acid, and *p*-nitrophenyl-β-D-glucopyranoside were purchased from Sigma-Aldrich Co. (USA), isoliquiritin and isoliquiritigenin from ChemFaces (China), and liquiritin apioside and isoliquiritin apioside from Chengdu Biopurify Phytochemicals Ltd. (China). All HPLC-grade solvents were purchased from Fisher Scientific Korea Ltd. (Korea). Extraction solution and methanol were purchased from Samchun Pure Chemical (Korea). Potato dextrose agar (PDA) was purchased from Acumedia Inc.(USA), and glucose from Duksan Pure Chemicals (Korea). Yeast extract-peptone-dextrose and yeast nitrogen base media were purchased from Sigma-Aldrich Co. *G. uralensis* base media were composed of 2.0 5.0% *G. uralensis* extracts and 2% glucose. Licorice (*G. uralensis*) was purchased from a regional market in Chungcheongbuk-do, Korea. Licorice extracts were prepared by adding 5 L of distilled water to 1.2 kg of licorice, followed by boiling at 95oC for 5 h. The extraction supernatant recovered by filtration with a Whatman filter paper No. 1 (UK) was concentrated to a solid content of 15% using a vacuum evaporator.

### Strains and Growth Conditions

Thirteen *Monascus* strains that had been isolated from *Monascus* fermentation products were used [19, 20]. In addition, four type strains, *Monascus pilosus* KCCM 60084, *Monascus purpureus* KCCM 60016, *Monascus ruber* KCTC 6122, and *Monascus kaoliang* KCCM 60154, obtained from the Korea Culture Center of Microorganisms (KCCM, Korea) or the Korean Collection for Type Cultures (KCTC, Korea), were used in the initial screening (Table 1).

Spores were harvested using a 0.85% saline buffer after culturing each of the strains on PDA plates for 5 days at 30°C. Spores were inoculated into 50 ml of *G. uralensis* base media at 1.0×10^6^ spore/ml. A 2% or 5% licorice medium was used for the initial screening or licorice fermentation. All broth cultures were incubated for 3 days (initial screening) or 10 days (fermentation) at 30°C with constant shaking at 200 rpm. The culture broth was freeze-dried using a freeze-dryer (FDU-1200, EYELA, Japan) for the analysis of fermentation products.

For an in vivo ER binding assay, the yeasts harboring pRR-ERβ-5Z (plasmid #23062) or pRR-ERα-5Z (plasmid #23061) were constructed by transforming each plasmid into *Saccharomyces cerevisiae* W300a obtained from Prof. S. J. Park (Yonsei University, Seoul, Korea) [11, 31]. Both plasmids were purchased from Addgene (USA). Yeast transformation was carried out using an MP BIO Alkali-Cation Yeast Transformation Kit (MP Biomedicals, USA) according to the kit protocol. Yeast was routinely cultured and maintained on a minimal media [11].

### Quantitative Analysis of Monacolin K, Citrinin, Liquiritin, Liquiritin Apioside, Liquiritigenin, Isoliquiritin, Isoliquiritin Apioside, and Isoliquiritigenin

Quantitative analyses of monacolin K and citrinin were carried out using HPLC as described previously for the analysis of *Monascus*-fermented red ginseng [14]. Liquiritin, isoliquiritin, liquiritigenin, isoliquiritigenin, liquiritin apioside and isoliquiritin apioside were also analyzed quantitatively [21] using an Agilent HPLC equipped with a HiQ sil C18W column (250 × 4.6 mm, particle size 5 μm, pore size 120 Å; Kya Tech Corporation, Japan). For each chemical analysis, 50 mg of a sample was extracted using 500 μl of methyl alcohol for 40 min with gentle shaking. All extracted samples were filtered using a 0.45-μl syringe filter (Life Sciences, USA). For the liquiritin, liquiritin apioside, isoliquiritin and isoliquiritn apioside HPLC analyses, samples (20 μl) were separated using an acetonitrile gradient mobile phase (20%acetonitrile for 10 min for the initial run, followed by 20–40%gradient for 2 min, 40% for 13 min, 40 20% gradient for 2 min, and 20% for 23 min) at a flow rate of 0.5 ml/min. Signals were detected using a UV detector (Agilent 1260 Infinity LC, Agilent Technologies, USA) at a wavelength of 254 and 370 nm for liquiritin and liquiritin apioside and for isoliquiritin and isoliquiritin apioside, respectively. For the liquiritigenin and isoliquiritigenin HPLC analyses, samples (20 μl) were separated using an acetonitrile gradient mobile phase (10–30% acetonitrile gradient for 10 min for the initial run, followed by 30–50%gradient for 35 min, 50–70% gradient for 10 min, 70–75% gradient for 10 min, 75–10% for 10 min, and 10% for 10 min) at a flow rate of 1 ml/min. Signals were detected at a wavelength of 274-nm and 370-nm wavelengths for liquiritigenin and for isoliquiritigenin, respectively.

### β-Glucosidase Assay

β-Glucosidase in the culture broth was measured as described previously with minor modifications [14]. Substrate solution was prepared by dissolving *p*-nitrophenyl-β-D-glucopyranoside in a 50 mM potassium phosphate buffer. Five hundred microliters of the aliquot was stored at –20°C until use. Two hundred microliters of supernatant from the culture broths was centrifuged at 13,750 ×g for 10 min, mixed with 500 μl of preheated substrate solution, and then incubated at 30°C for 30 min. Released *p*-nitrophenol was measured using a spectrophotometer at 405 nm. One unit of β-glucosidase activity was defined as the release of 1 μmole of *p*-nitrophenol per min.

### Yeast Estrogen Binding Assay

The ERα and β binding assays were performed using *S. cerevisiae* harboring pRR-ERα-5Z or pRR-ER β -5Z [31] as described by Fox (2008) [11]. Yeast was pre-cultured in a minimal medium for approximately 24 h until the optical density (OD) at 600 nm exceeded 1. Subsequently, culture was diluted to OD = 0.4 with a fresh medium. Diluted yeast culture (200 μl) and 5 μl of *Monascus*-fermented licorice extract were transferred to a 96-well plate. The plate was incubated for 18 h at 30°C. Culture (50 μl) was mixed with a 200 μl of Lac-Z buffer in a new 96-well plate, and then incubated at 30°C for 15 min. Color development was monitored at 405 nm. The ER binding activity was expressed as % relative absorbance to the 17 β-estradiol treatment whose maximum absorbance was set to 100% [11].

## Results and Discussion

### Screening for *Monascus* Strains Suitable for the Bioconversion of Liquiritin and Isoliquiritin in Licorice (*G. uralensis*)

**Fig. 1:**
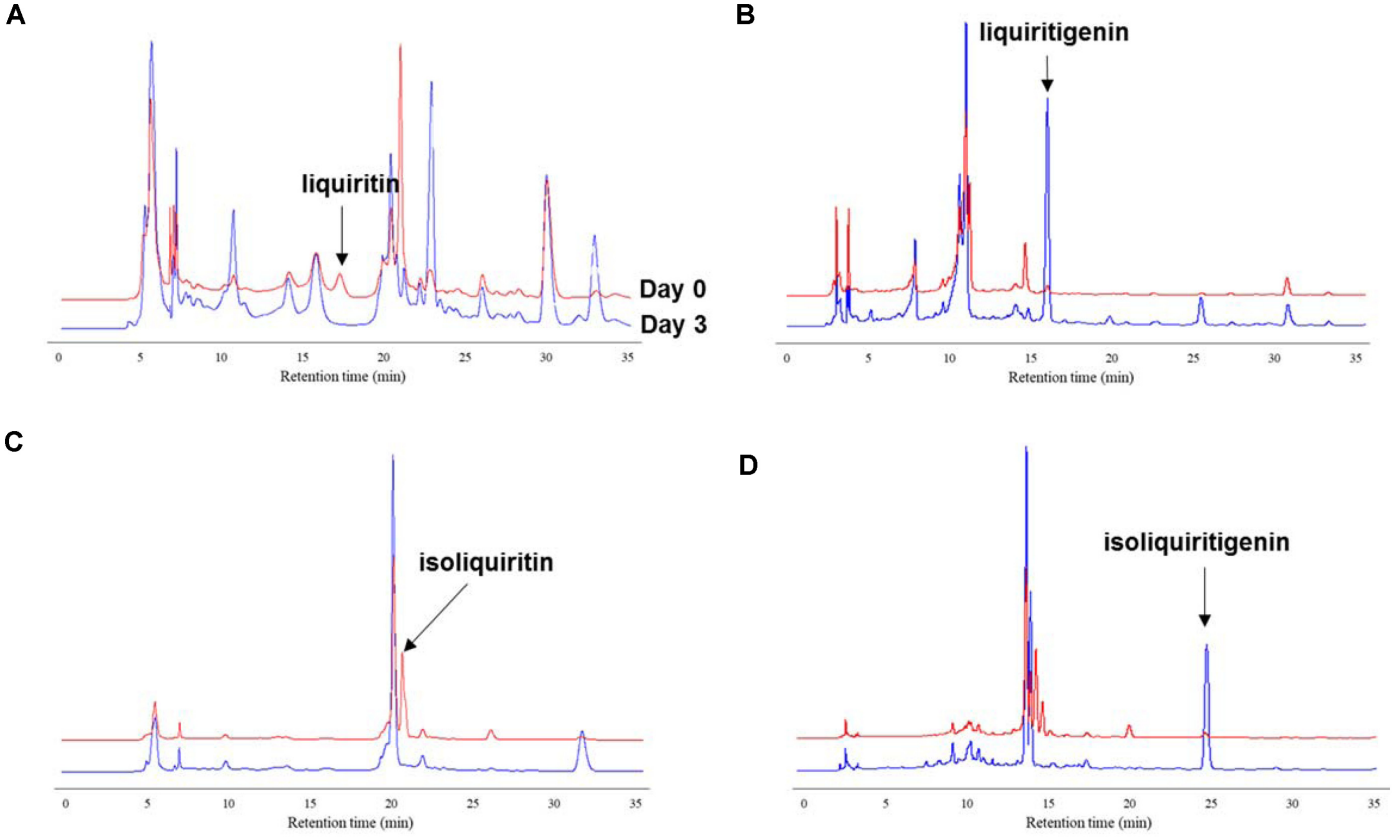
HPLC chromatograms of liquiritin (**A**), liquiritigenin (**B**), isoliquiritin (**C**), and isoliquiritigenin (**D**).

We have reported previously that *Monascus* spp. can convert glycosides into their corresponding aglycones [14, 24]. In the present study, we attempted to bioconvert the glycosidic components in licorice, such as liquiritin and liquiritin apioside, into liquiritigenin, a well-known selective agonist for ERβ. First, we tested and screened for *Monascus* strains suitable for the deglycosylation of liquiritin and isoliquiritin in licorice. We have previously isolated 17 *Monascus* strains from *Monascus*-fermented products [19, 20]. Each strain exhibited the strain-specific characteristics with regard to enzyme and secondary metabolite production, such as monacolin K, pigments, and citrinin, under different fermentation substrates [14, 24]. Therefore, the strains were evaluated for the deglycosylation of liquiritin and isoliquiritin and for the production of monacolin K in licorice media. Liquiritin and isoliquiritin in licorice were all converted into the corresponding deglycosylated compounds by fermentation with *Monascus* spp. The liquiritin peak (retention time 17.2 min) disappeared (Fig. 1A), and the liquiritigenin peak (retention time 15.8 min) emerged (Fig. 1B) after 3 days of fermentation. Similarly, the isoliquiritin peak (retention time 20.9 min) disappeared (Fig. 1C) and the isoliquiritigenin peak (retention time 24.8 min) was observed (Fig. 1D) after 3 days of fermentation.

The liquiritigenin and isoliquiritigenin contents in the freeze-dried *Monascus*-fermented licorice ranged from 0.37 to 3.94 mg/g and from 0.13 to 0.68 mg/g, respectively (Table 1). *M. pilosus* U exhibited the highest bioconversion activities for liquiritigenin and isoliquiritigenin, which were observed at 3.94 and 0.68 mg/g, respectively, and was followed by *M. pilosus* KMU108, *M. pilosus* I, and *M. albidulus* N (Table 1). On the other hand, most strains produced monacolin K at various levels, ranging from 130.9 to 3,744.1 mg/g, and *M. pilosus* A, *M. purpureus* B, *M. pilosus* K, and *M. albidulus* N produced over 500 mg/g of monacolin K, which is the minimum level to be claimed as a functional food in the case of *Monascus*-fermented red yeast rice (2014 Health Functional Food Code, Ministry of Food and Drug Safety (MFDS), Korea). In addition, most of the strains produced citrinin at very low concentrations, and citrinin was not detected in *M. albidulus* N-fermented licorice (data not shown). Since citrinin is a nephrotoxic and hepatotoxic fungal toxin [4, 22], the selection of a strain that produces little or no citrinin is very critical for functional food materials. The level of citrinin production in *Monascus* spp. is known to be substrate dependent [35]. Therefore, *M. albidulus* N was selected as the strain suitable for the bioconversion of liquiritin and isoliquiritin in licorice. It has been identified previously as *M. albidulus* [19, 20] and was designated as *M. albidulus* KMU112.

### Bioconversion of Liquiritin and Isoliquiritin in Licorice during Fermentation with *M. albidulus* KMU112

**Fig. 2:**
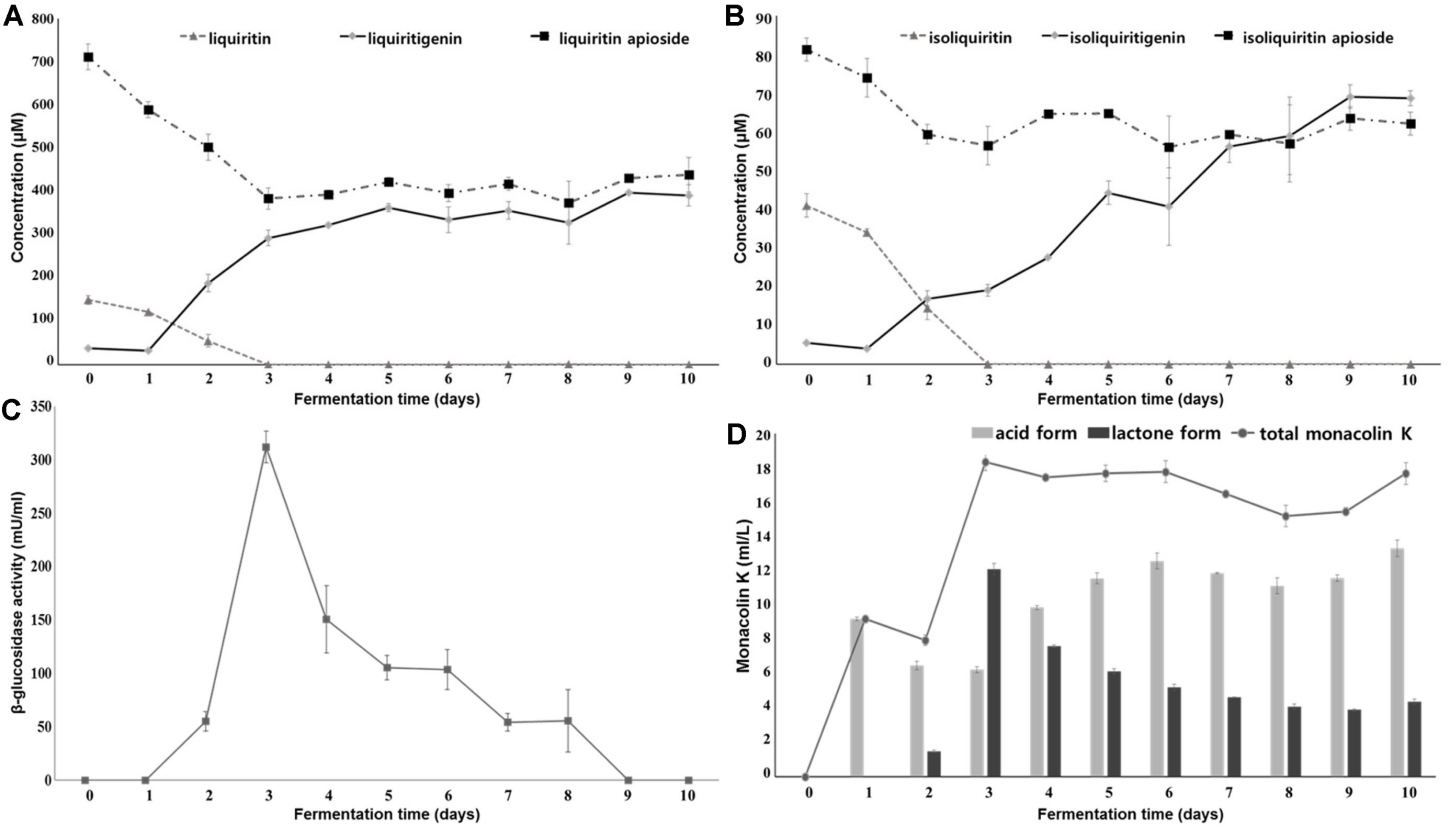
Bioconversion of liquiritin/liquiritin apioside and isoliquiritin/isoliquiritin apioside, β-glucosidase activity, and monacolin K production during licorice fermentation. (**A**) Bioconversion of liquiritin and liquiritin apioside to liquiritigenin; (**B**) bioconversion of isoliquiritin and isoliquiritin apioside to isoliquiritigenin; (**C**) β-glucosidase activity; (**D**) monacolin K production.

**Fig. 3:**
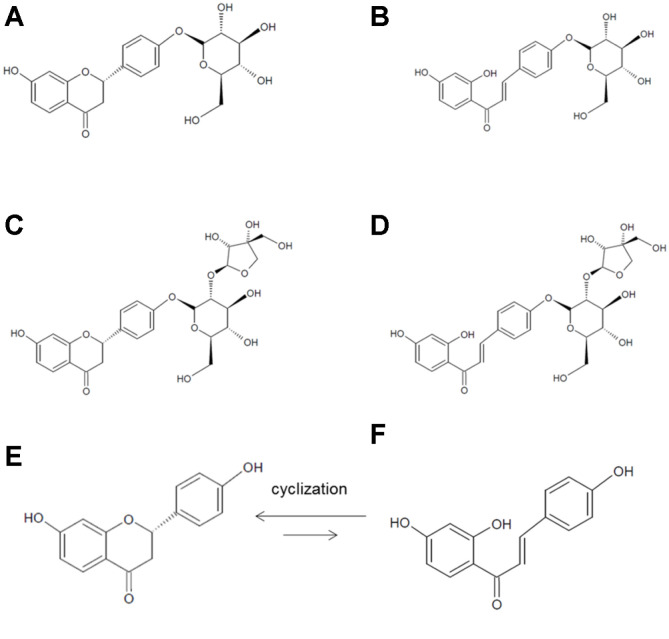
Chemical structures of liquiritin (**A**), isoliquiritin (**B**), liquiritin apioside (**C**), isoliquiritin apioside (**D**), liquiritigenin (**E**), and isoliquiritigenin (**F**).

The liquiritin concentration was 149.64 μM, whereas that of liquiritigenin was very low at 38.03 μM in a 5% preculture licorice medium (Fig. 2A), suggesting that most of liquiritin exists in a glycosylated form in licorice. After 3 days of fermentation, liquiritin was no longer detected, and liquiritigenin concentrations increased to 322.63 μM on day 4 of fermentation and reached a maximum of 397.75 μM on day 9 of fermentation (Fig. 2A). Such an unexpected increase in the concentration of liquiritigenin suggested the existence of liquiritin analogs in licorice and their bioconversion into liquiritigenin during *Monascus* fermentation. Liquiritin apioside is another liquiritin glycoside (Fig. 3), observed at 1.27% on average in *G. uralensis* and more than 1% in various licorice species [21]. Consequently, some of liquiritigenin could have been derived from liquiritin apioside, and the concentrations were 712.05 μM before fermentation and 383.82 μM on day 3 of fermentation, respectively (Fig. 2A).

The concentrations of isoliquiritin and isoliquiritigenin were 40.99 μM and 5.53 μM, respectively, in the licorice medium, suggesting that a glycolylated form has higher concentration than an aglycoylated form, such as liquiritin/liquiritgenin. Isoliquiritin was also not detected after 3 days of fermentation, and the concentrations of isoliquiritigenin increased steadily during the fermentation period and reached a maximum of 69.14 μM on day 9. Some of the isoliquiritigenin could also be derived from isoliquiritin apioside, which is an isoliquiritin glycoside (Fig. 3). Such bioconversion was supported by the 30.63% reduction in isoliquiritin apioside from 81.32 μM to 56.41 μM on the day 3 culture (Fig. 2B).

Liquiritin and isoliquiritin are bioconverted into liquiritigenin and isoliquiritigenin by β-glucosidase [2, 45]. The β-glucosidase activity was detected in fermented licorice and reached a maximum of 312.02 mU/ml after 3 days of fermentation, suggesting that liquiritin and isoliquiritin of licorice were bioconverted by β-glucosidase in *M. albidulus* KMU112 (Fig. 2C). Conversely, the concentrations of liquiritin apioside and isoliquiritin apioside decreased until day 3 and then maintained a constant concentration, and the decreases represented 46.10% and 30.63% reductions from the initial concentrations, respectively. The reason for the partial bioconversion of liquiritin/isoliquiritin apiosides remains unclear; however, apiosides seem to be converted in different ways compared with liquiritin/isoliquiritin. According to Sato (2007) [37], liquiritin/liquiritin apioside and isoliquiritin/isoliquiritin apioside were bioconverted completely by naringinase, which has both α-L-rhamnosidase and β-glucosidase functions. Therefore, liquiritin apioside and isoliquiritin apioside could be bioconverted by enzymes other than β-glucosidase [37].

Since liquiritigenin and isoliquiritigenin can be interconverted naturally [38], the final liquiritigenin and isoliquiritigenin concentrations may not be proportional to each bioconversion. In summary, glycosylated forms of liquiritin/isoliquiritin and liquiritin apioside/isoliquiritin apioside were successfully biotransformed into their corresponding deglycosylated forms by fermentation.

### Monacolin K Production during Licorice Fermentation by *M. albidulus* KMU112

Total monacolin K was detected at 18.35 mg/L on day 3 of culture, and the concentration was maintained until day 10 of fermentation (Fig. 2D). The active monacolin K (acid form) was produced mainly during the early fermentation period (9.21 mg/l on day 1), and then the concentration increased gradually up to the maximum level on day 6 of fermentation (12.58 mg/l). Conversely, the inactive monacolin K (lactone form) was detected after day 2, and reached a maximum (12.11 mg/l) on day 3, followed by a gradual decrease. The pH of the culture broth decreased until day 3 of fermentation and then increased after, suggesting that an increase in the active acid form after day 3 was in part derived from the inactive lactone form due to change in pH [28]. The active and inactive monacolin K concentrations in the freeze-dried powder of the 10-day culture were 551.03 mg/kg and 180.55 mg/kg, respectively. Monacolin K production has been demonstrated to be affected by substrates. The monacolin K concentrations in *Monascus*-fermented soybean, fortified mugwort, or ginseng were 1,366, 2,219, and 3,089 mg/kg [14, 24, 25]. In addition, the concentration of a substrate influences monacolin K production so that monacolin K yield was generally higher under lower substrate concentrations [41]. In the present study, 5% licorice medium instead of 2% licorice medium was used for strain screening to increase liquiritigenin and isoliquiritingenin concentrations. The amounts of active monacolin K in fermented licorice exceed the monacolin K standard concentrations in the *Monascus*-fermented red yeast rice functional food (2014 Health Functional Food Code, MFDS, Korea). Therefore, the fermented licorice could prevent the cardiovascular diseases associated with the adverse side effects of the female climacteric syndrome, which is an additional benefit of fermented licorice.

### In Vivo ER Binding Assay of *Monascus*-Fermented Licorice Extracts

**Fig. 4:**
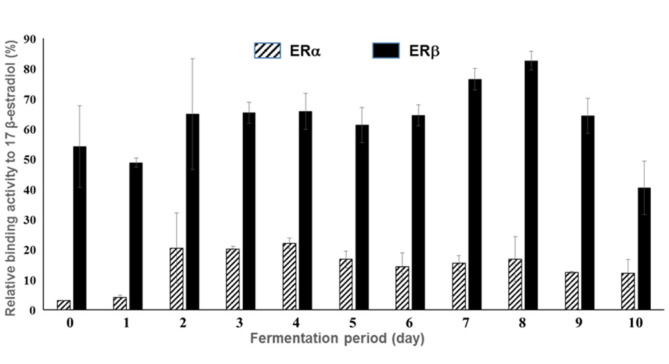
ER binding activities of the licorice fermentation extracts. The ERα and ERβ-binding activities of licorice extracts were evaluated before and after fermentation using an in vivo assay system. ER binding activity was expressed as % relative absorbance to the 17 β- estradiol treatment whose maximum absorbance was set to 100%.

The ERα and ERβ binding activities of the *Monascus*-fermented licorice extracts were evaluated using recombinant yeasts that contained the ERα or ERβ gene fused to a β-galactosidase reporter gene. The ERα and ERβ binding activities of the extracts of pre-fermented licorice media were 3.04% and 54.06% that of the positive control, 17 β-estradiol, respectively (Fig. 4), which is consistent with the findings of a study where licorice exhibited higher ERβ binding activity compared with ERα binding activity [42]. Both ERα and ERβ binding activities increased by fermentation with *M. albidulus*. On day 4 of fermentation, the ERα binding activity reached to the maximum level, which was 22.0% that of the positive control. Conversely, the ERβ binding activity increased up to 82.54% with the extracts of the 8-day fermented licorice. However, the increase in ER binding activity was not proportional to the liquiritigenin and isoliquiritigenin concentrations, suggesting the involvement of other components in ER binding. Licorice contains not only estrogenic agonists but also estrogenic antagonists [5].

The ERα and ERβ binding activity of licorice has been reported using the MCF-7 cells; however, the binding activity was reported to be low compared with the positive control [5, 30]. In the present study, we demonstrate that fermentation with *Monascus* could considerably increase the ERα and ERβ binding activities of licorice. Particularly, the ERβ binding activity was almost equivalent to that of the positive control. Such an increase in the selective binding capacity of fermented licorice to ERβ could be due to increased liquiritigenin concentrations in the course of fermentation. Liquiritigenin binds selectively to ERβ [30].

According to van Patten (2002), a very high binding activity for ERα is associated with phytoestrogen-induced side effects, such as breast cancer [43]. The most commonly used phytoestrogens, soybean isoflavones, have a high binding affinity for ERα. For example, genistein exhibits approximately 60% ERα binding ability [6]. In recent years, the Japanese Food Safety Commission has limited the daily intake of isoflavone to less than 30 mg/kg in a specialized diet, which was also adopted by MFDS in Korea [1, 13, 15]. The fermented licorice in the present study exhibited an ER binding activity, preferentially to ERβ, of up to 82.5%. Therefore, fermented licorice could have an advantage over other phytoestrogens in the alleviation of menopause symptoms with low side effects.

Market demand for women's menopausal remedies is on the rise due to growing interest in women’s health. In the present study, *Monascus*-fermented licorice contained high levels of liquiritigenin and monacolin K and exhibited high ERβ-selective binding activity. Therefore, *Monascus*-fermented licorice is a promising material that could be applied in the prevention and treatment of menopausal syndrome with or without minimal side effects.

## Figures and Tables

**Table 1:** Contents of liquiritigenin and isoliquiritigenin, and monacolin K production in licorice fermented with *Monascus* strains.

Strain	Origin	Fermented solid contents (%)	Monacolin K (mg/kg)			Liquiritigenin (mg/g)	Isoliquiritigenin (mg/g)

			Acid form	Lactone form	Total		
*M. pilosus* A	Red mold rice, Korea	48.90 ± 2.37	834.06 ± 176.20	2,198.29 ± 188.64	3,032.35 ± 247.99	0.74 ± 0.16	0.39 ± 0.07
*M. purpureus* B	Red mold rice, Korea	56.26 ± 4.12	136.86 ± 27.48	389.72 ± 44.26	526.58 ± 57.89	1.73 ± 0.22	0.22 ± 0.06
*M. pilosus* KMU106	Red mold rice, Korea	54.59 ± 2.37	142.40 ± 37.41	ND^c^	142.4 ± 37.41	2.66 ± 0.31	0.42 ± 0.08
*M. pilosus* D	Red mold rice, Korea	52.48 ± 2.16	142.79 ± 11.16	ND	142.79 ± 11.16	0.74 ± 0.11	0.31 ± 0.12
*M. pilosus* F	Red mold rice, Korea	50.13 ± 5.37	139.28 ± 18.14	ND	139.28 ± 18.14	0.61 ± 0.14	0.13 ± 0.04
*M. pilosus* KMU108	Red mold rice, Korea	53.31 ± 4.18	147.45 ± 25.18	ND	147.45 ± 25.18	3.83 ± 0.71	0.60 ± 0.04
*M. pilosus* I	Red mold rice, Korea	52.43 ± 3.76	146.46 ± 31.43	ND	146.46 ± 31.43	3.60 ± 0.34	0.63 ± 0.05
*M. pilosus* K	Red mold rice powder, China	45.96 ± 3.72	807.37 ± 90.11	2,426.45 ± 213.86	3,233.82 ± 300.41	1.87 ± 0.18	0.34 ± 0.05
*M. pilosus* M	Red mold rice powder, China	52.19 ± 6.54	151.09 ± 21.87	ND	151.09 ± 21.87	3.18 ± 0.26	0.53 ± 0.12
*M. albidulus* N	Red mold rice powder, China	44.82 ± 3.11	874.01 ± 48.64	2,870.10 ± 128.19	3,744.11 ± 166.87	3.22 ± 0.31	0.40 ± 0.08
*M. pilosus* O	Red mold rice powder, China	52.67 ± 1.48	130.90 ± 12.89	ND	130.9 ± 12.89	3.14 ± 0.48	0.64 ± 0.08
*M. pilosus* T	Red mold rice powder, China	54.22 ± 2.86	130.90 ± 6.48	ND	130.9 ± 6.48	2.24 ± 0.29	0.67 ± 0.11
*M. pilosus* U	Red mold rice powder, China	51.68 ± 5.12	140.58 ± 17.78	ND	140.58 ± 17.78	3.94 ± 0.34	0.68 ± 0.16
*M. pilosus* KCCM60084	KCCM^a^	53.33 ± 3.42	142.64 ± 22.68	ND	142.64 ± 22.68	3.44 ± 0.16	0.63 ± 0.07
*M. purpureus* KCCM60016	KCCM	55.30 ± 3.76	144.77 ± 17.64	39.18 ± 2.84	183.95 ± 20.68	2.19 ± 0.15	0.38 ± 0.08
*M. ruber* KCTC6122	KCTC^b^	57.38 ± 4.29	145.13 ± 7.66	225.99 ± 29.87	371.12 ± 38.04	0.37 ± 0.08	0.16 ± 0.08
*M. kaoliang* KCCM60154	KCCM	53.13 ± 4.86	152.62 ± 10.04	92.59 ± 11.64	245.21 ± 20.80	3.18 ± 0.24	0.52 ± 0.12

^a^KCCM, Korea Culture Center of Microorganisms.

^b^KCTC, Korean Collection for Type Cultures.

^c^ND, not detected.

Fermentation was carried out at 30°C with shaking at 200 rpm for 150 h in a 2% licorice medium.
